# Bioinspired Antimicrobial Strategy: An Extremophile Deep Sea Peptide to Combat Cystic Fibrosis Infections Caused by *Pseudomonas aeruginosa* and *Staphylococcus aureus*

**DOI:** 10.3390/md24050164

**Published:** 2026-05-05

**Authors:** Céline Boidin-Wichlacz, Marc Maresca, Teddy Grandjean, Axelle Grandé, Orane Huchez, Katy Jeannot, Rémi Desmet, Benoît Snella, Nicolas Vidal, Laure Genet, Stéphanie Caby, Magalie Sénéchal, Sophie Guillier, Fabienne Ripoll-Neulat, Oleg Melnyk, Muriel Pichavant, Aurélie Tasiemski

**Affiliations:** 1 Univ. Lille, CNRS, Inserm, CHU Lille, Institut Pasteur de Lille, U1019-UMR 9017-CIIL—Center for Infection and Immunity of Lille, F-59000 Lille, France; celine.wichlacz@univ-lille.fr (C.B.-W.); teddy.grandjean@univ-lille.fr (T.G.); axelle.grande@inserm.fr (A.G.); orane.cheddad@cnrs.fr (O.H.); remi.desmet@ibl.cnrs.fr (R.D.); benoit.snella@cnrs.fr (B.S.); stephanie.caby@pasteur-lille.fr (S.C.); magalie.senechal@cnrs.fr (M.S.); oleg.melnyk@ibl.cnrs.fr (O.M.); muriel.pichavant@pasteur-lille.fr (M.P.); 2 Aix Marseille Univ, CNRS, Centrale Med, ISM2, F-13013 Marseille, France; m.maresca@univ-amu.fr; 3 Université Marie et Louis Pasteur, CHU Besançon, CNRS, Chrono-Environnement (UMR 6249), F-25000 Besançon, France; katy.jeannot@univ-fcomte.fr; 4 Yelen Analytics, 10 bd tempête, F-13820 Ensues La Redonne, France; nicolas.vidal@yelen-analytics.com; 5 Institut de Recherche Biomédicale des Armées (IRBA)-UMR-MD1-Inserm 1261, F-91220 Brétigny-Sur-Orge, France; laure.genet@intradef.gouv.fr (L.G.); sophie.guillier@intradef.gouv.fr (S.G.); fabienne.ripoll@intradef.gouv.fr (F.R.-N.); 6 Aix Marseille Univ, INSERM, SSA, MCT, F-13385 Marseille, France

**Keywords:** antibiotic, resistance, antibiofilm, extremophile, worms, biomimicry, redox, lung

## Abstract

Cystic fibrosis (CF)-associated lung infections caused by *Pseudomonas aeruginosa* (*P. aeruginosa*) and *Staphylococcus aureus* (*S. aureus*) remain difficult to treat due to multidrug resistance and the redox instability of the pulmonary environment, which can impair antibiotic efficacy. In this study, we investigated alvinellacin (ALV), a disulfide-stabilized β-hairpin antimicrobial peptide (AMP) derived from the deep-sea polychaete *Alvinella pompejana (A. pompejana*), as a potential therapeutic agent naturally adapted to redox-fluctuating conditions. The antibacterial and antibiofilm activities of ALV were evaluated against multidrug-resistant (MDR) clinical isolates under CF-like reducing conditions (6 mM dithiothreitol (DTT)). Circular dichroism (CD) analysis showed that DTT did not alter the β-hairpin secondary structure of ALV, supporting its structural stability in CF-like environments. Mechanistic analyses included pore-forming assay, membrane interaction studies, scanning electron microscopy (SEM), lipid-binding assays, cytotoxicity testing, and resistance induction assays, while *in vivo* efficacy was assessed using the *Galleria mellonella* infection model. ALV demonstrated strong bactericidal activity that was maintained in the presence of NaCl or human serum. ALV did not induce bacterial resistance and effectively inhibited early-stage biofilm formation and disrupted preformed biofilms, including those of the clinical isolate, even under reducing conditions. The peptide showed selective permeabilization of bacterial membranes linked to its stronger affinity for bacterial membrane lipids and negligible interaction with host-like membranes, with no observed cytotoxicity. *In vivo*, ALV significantly improved survival in infected larvae. These findings highlight ALV as a promising redox-resilient antimicrobial candidate for treating MDR CF lung infections.

## 1. Introduction

Chronic bacterial infections exemplify how microenvironmental stress can transform microbial colonization into a lifelong therapeutic challenge, particularly in CF where redox imbalance and biofilm formation undermine antibiotic efficacy. CF is a life-shortening, monogenic disorder caused by mutations in the CFTR gene, leading to defective chloride transport, impaired mucociliary clearance, and the accumulation of dehydrated, viscous mucus in the airways. This pathological environment promotes chronic neutrophilic inflammation and persistent bacterial infections. Among the predominant pathogens colonizing the CF airway are *P. aeruginosa* and *S. aureus*, along with other opportunistic and emerging bacteria [1]. These pathogens adapt to intensive and prolonged antibiotic exposure, forming resilient biofilms [2] and developing multidrug resistance, which severely limits therapeutic options. Consequently, there is an urgent need for antimicrobials with novel modes of action, enhanced stability in pulmonary secretions and minimal propensity to select for resistance.

Redox dysregulation has emerged as a critical determinant of infection persistence and antibiotic performance in CF airways. Multiple studies have shown that bacterial infections in CF induce intracellular and extracellular shifts in redox homeostasis. In healthy individuals, the airway surface liquid maintains an oxidizing redox potential (+100 to +200 mV) and near-neutral pH (7.0–7.5), optimal for mucosal defense [3]. In contrast, CF airway secretions are acidic (pH 5.0–6.5) and strongly reducing (−200 to −300 mV) due to hypoxia, neutrophilic inflammation, and the accumulation of reduced thiols such as glutathione and cysteine. Such a redox-imbalanced microenvironment alters the activity of both host-derived and therapeutic bioactive molecules, thereby promoting bacterial persistence [4,5]. In the context of CF lung infections, colistin (CST), a cyclic AMP, is among the few antibiotics that retain antibacterial efficacy under acidic and reducing microenvironments characteristic of the CF airway [6]. In contrast, β-lactam antibiotics, including imipenem (IPM)and ceftazidime, demonstrate significantly reduced activity under these conditions, with both acidic pH and redox stress compromising their bactericidal functions [7]. This underscores the importance of identifying novel antimicrobials that do not induce resistance and are not cytotoxic while maintaining their efficacy in fluctuating redox environments such as the CF lung.

Interestingly, while arising in very different contexts, the chemical imbalance observed in the airways of individuals with CF provides a useful analogy to chemical fluctuations naturally occurring in hydrothermal vent ecosystems [8]. In both systems, a steep gradient exists between oxidizing zones rich in oxygen and reducing zones dominated by reactive species such as sulfides. In CF lungs, thick mucus layers and persistent bacterial biofilms lead to the local depletion of oxygen, resulting in the formation of hypoxic or anoxic niches with redox potential (Eh) values dropping from +400 mV to below 0 mV [9,10]. While not directly comparable, this redox stratification provides a useful analogy to that described for *A. pompejana*’s tube [11]. *A. pompejana*, is a deep-sea worm endemic to hydrothermal vents along the East Pacific Rise. It inhabits a self-constructed tube situated at the interface between cold, oxygenated seawater and hot, anoxic, sulfur-rich vent fluids where the redox potential may shift by over 600 mV—from +450 mV to below −300 mV across less than 1 cm [11,12]. To thrive in chemically unstable environments such as hydrothermal vents, resident species have evolved specialized adaptive strategies to preserve protein structure and function [13]. In *A. pompejana*, one such adaptation is the production of ALV, a β-hairpin cationic AMP stabilized by two intramolecular disulfide bonds [14]. In contrast, AMP analogs produced by temperate or polar annelid species contain either one single disulfide bond or none at all [15]. Remarkably, ALV retains its bactericidal activity against hydrothermal *Pseudomonas* strains related to *P. aeruginosa*, the primary pathogen in CF, even under high salt concentrations and across a wide pH range [15], conditions that typically inactivate many AMPs [16] and antibiotics [17,18]. The salt-induced inactivation of host defense peptides represents a significant challenge in CF patients [19,20]. In this context, ALV’s capacity to rapidly and efficiently kill bacteria such as *Pseudomonas* spp. in high-salt environments highlights its potential for drug development.

Based on the salt and pH adaptation of ALV to hydrothermal vents [15,21], we hypothesized that this extremophile AMP has also evolved to be redox-resilient. To test this, we evaluated the antibacterial and antibiofilm activities of ALV against the Gram-negative *P. aeruginosa* and the Gram-positive *S. aureus*, including MDR clinical isolates, under conditions mimicking the redox microenvironment of CF-infected airways (+6 mM DTT, corresponding to −300 mV). In parallel, we investigated the structural stability of ALV in the presence of DTT by CD spectroscopy to determine whether reducing conditions affect its secondary structure in tandem with the observed biological activities. By examining its functional spectrum, mechanism of action, and in vivo protective efficacy against infection, we aimed to assess the potential of ALV for the development of novel antimicrobials effective in biofilm-rich respiratory settings.

## 2. Results

### 2.1. ALV Exerts Rapid and Potent Bactericidal Activity Against P. aeruginosa and S. aureus

**Figure 1:**
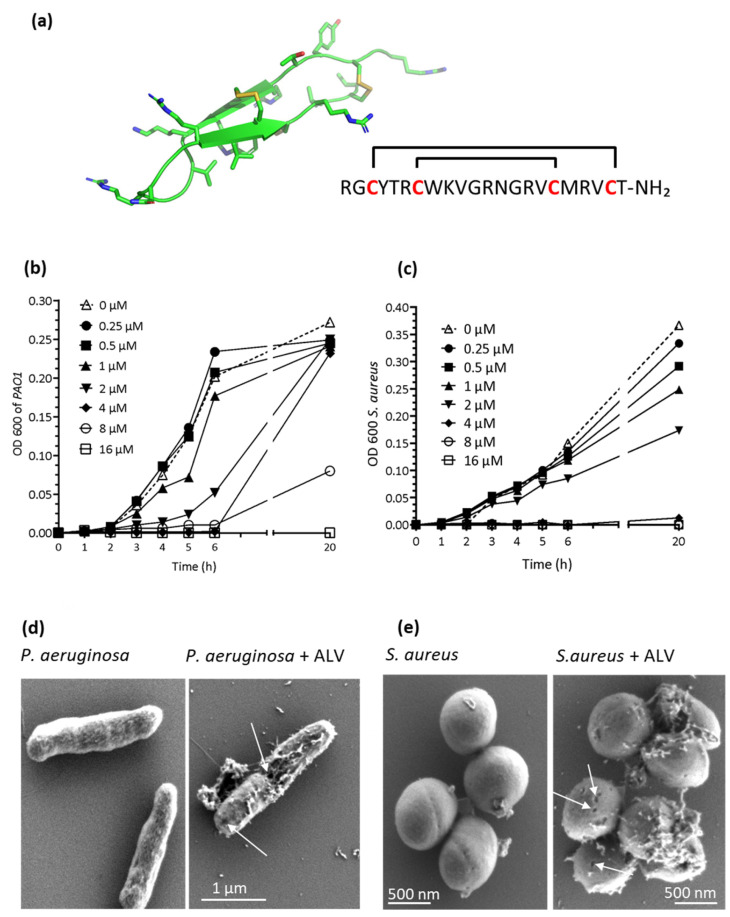
Structure and growth inhibition effects of ALV on *P. aeruginosa* and *S. aureus*. (**a**) Nuclear Magnetic Resonance (NMR) structure (pdb 2llr, hydrogens are omitted for clarity) [14] and amino acid sequence of ALV. (**b**,**c**) Effects of increasing concentration of ALV on the growth of *P. aeruginosa* (**b**) and *S. aureus* (**c**) over a 20-h incubation period. A break in the x-axis was introduced to improve data readability. (**d**,**e**) SEM images of *P. aeruginosa* (**d**) and *S. aureus* (**e**) following ALV treatment. Arrows indicate membrane damage and pore formation.

As shown in Table 1, ALV (Figure 1a) exhibited strong antibacterial activity at the µmolar range against both reference and clinical strains of *P. aeruginosa* and *S. aureus*. Lower concentrations were required to inhibit *P. aeruginosa* compared to *S. aureus*.

MIC and MBC values were identical for all tested strains, indicating a clear bactericidal effect. The impact of NaCl salt and human serum on ALV activity was then evaluated (Table 2). MIC and MBC values of ALV on *P. aeruginosa* and *S. aureus* were not affected or were weakly affected by NaCl at 150 mM (physiological concentration) or 300 mM (high concentration found in the pulmonary fluid of CF patients). Similarly, the incubation of ALV at 37 °C for 4 h with 50% human serum did not affect its activity.

Growth inhibition assays further demonstrated that ALV at MIC/MBC (4 µM for *P. aeruginosa* and 8 µM for *S. aureus*) values induced an immediate loss of bacterial growth upon exposure (Figure 1b,c).

**Figure 2:**
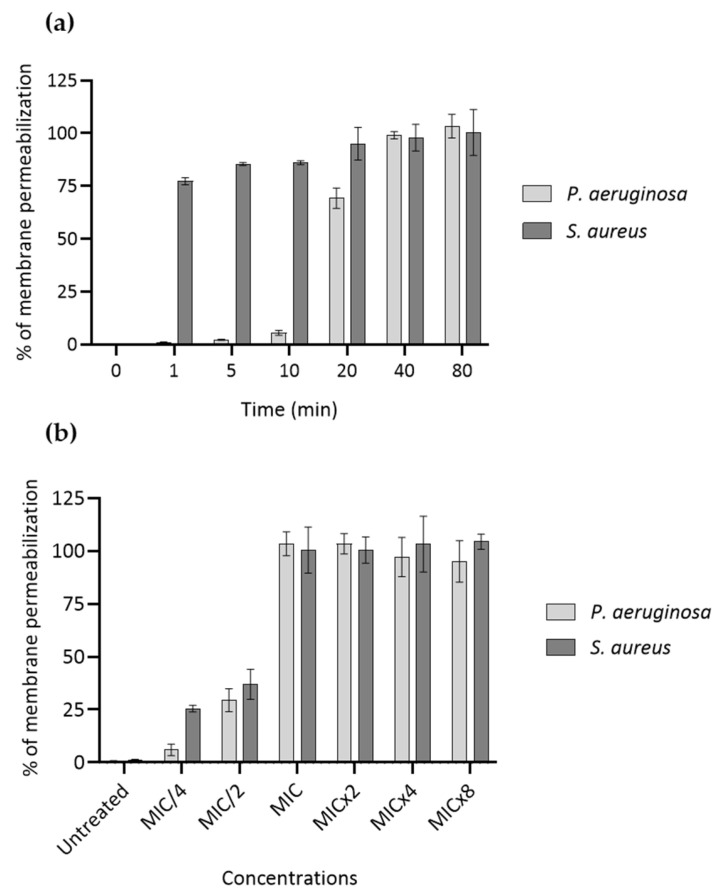
Membrane permeabilization effect of ALV against *P. aeruginosa* and *S. aureus*. (**a**) Dose-dependent effect at 80 min of exposure and (**b**) time-dependent at MIC of ALV on the membrane integrity of *P. aeruginosa* (PA01) and *S. aureus* (ATCC 29213). Membrane permeabilization was measured through determination of the release of intracellular ATP into culture media using a luciferase assay. Values are expressed as percentage of membrane permeabilization, with negative controls (untreated condition) corresponding to bacteria treated with vehicle alone and positive controls corresponding to 100% bacterial lysis using lysis buffer from the ATP assay kit. Results are expressed as means ± SD (*n* = 3).

SEM revealed pronounced morphological alterations consistent with pore formation on the bacterial surface (Figure 1d,e), supporting a membrane-disruptive mode of action of ALV in both bacterial species. Pore-forming activity of ALV was further investigated using an ATP release assay (Figure 2).

Dose-dependent assay after 80 min of incubation (Figure 2a) showed that ALV causes a total permeabilization of both *P. aeruginosa* and *S. aureus* at concentrations equal or superior to their MIC values, with concentrations corresponding to MIC/2 and MIC/4 causing only partial permeabilization (ranging from 5.9 to 36.9% of intracellular ATP release). Time-dependent assay at MIC (Figure 2b) demonstrated that the kinetics of membrane permeabilization caused by ALV is strain-dependent. Whereas membrane permeabilization of *S. aureus* is very fast (with 77.3% permeabilization at 1 min of exposure), pore formation is slower in *P. aeruginosa* (69.2% of permeabilization being obtained after 20 min of exposure).

### 2.2. ALV Exhibits Bactericidal and Antibiofilm Activities Against P. aeruginosa and S. aureus

**Figure 3:**
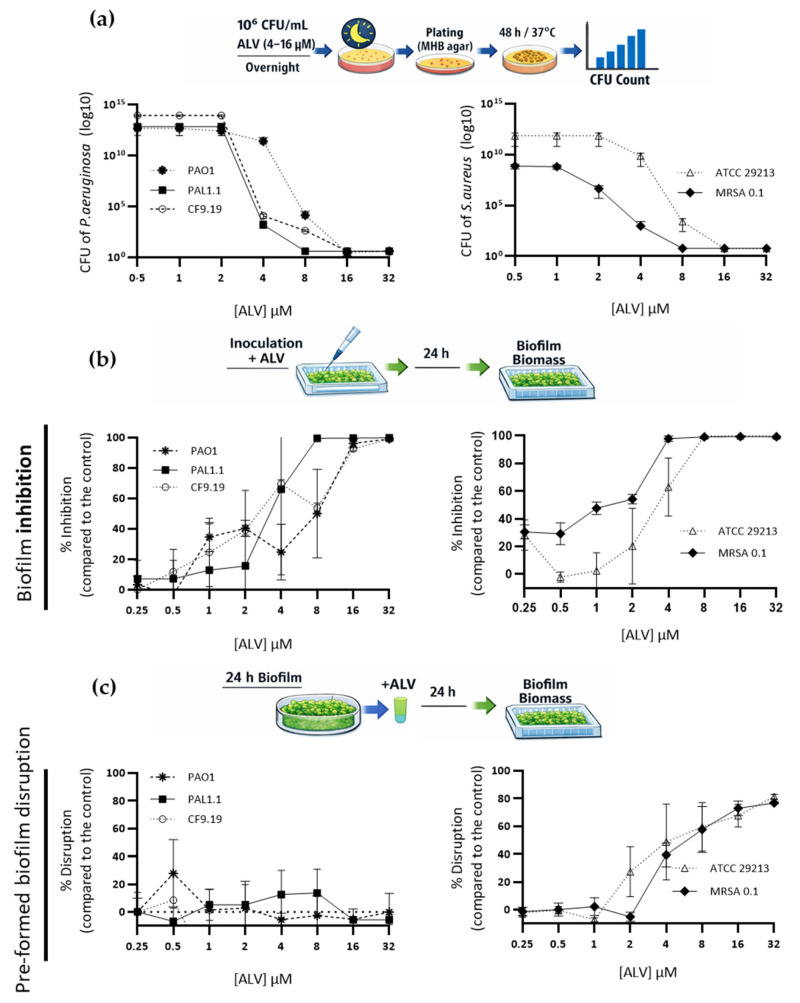
Dose-dependent antibacterial and antibiofilm activities of ALV against reference and clinical strains of *P. aeruginosa* (**left**) and *S. aureus* (**right**). The reference strains are PAO1 for *P. aeruginosa* and ATCC 29213 for *S. aureus*. (**a**) Dose-dependent killing curves following exposure to ALV. Bacterial survival was quantified by colony-forming unit (CFU) counting. Data are presented as log10 CFU/mL as a function of peptide concentration. Lines indicate the mean ± SD (*n* = 3). (**b**) Inhibition of biofilm formation by ALV is expressed as the percentage (%) relative to the untreated control. Each point represents an individual replicate (*n* = 3), and lines indicate the mean ± SD. (**c**) Disruption of preformed biofilms is expressed as the percentage (%) relative to the untreated control. Each point represents an individual replicate (*n* = 3), and lines indicate the mean ± SD.

Dose-dependent assays performed on both bacterial species demonstrated that ALV exerts a bactericidal effect against both clinical and reference strains (Figure 3a).

Exposure to different concentrations of ALV, including sub-MIC values (≤4 µM for *P. aeruginosa* and ≤8 µM for *S. aureus*), resulted in a marked inhibition of biofilm formation in both clinical (PAL1.1 and MRSA1) and reference (PAO1 and *S. aureus* ATCC 29213) strains (Figure 3b). Adding ALV to pre-formed *P. aeruginosa* biofilms has no significant effect, whereas 60–80% of pre-formed *S. aureus* biofilms are disrupted at MBC values (Figure 3c).

### 2.3. ALV Displays a Low Propensity to Induce Resistance Compared to Reference Antibiotics

**Figure 4:**
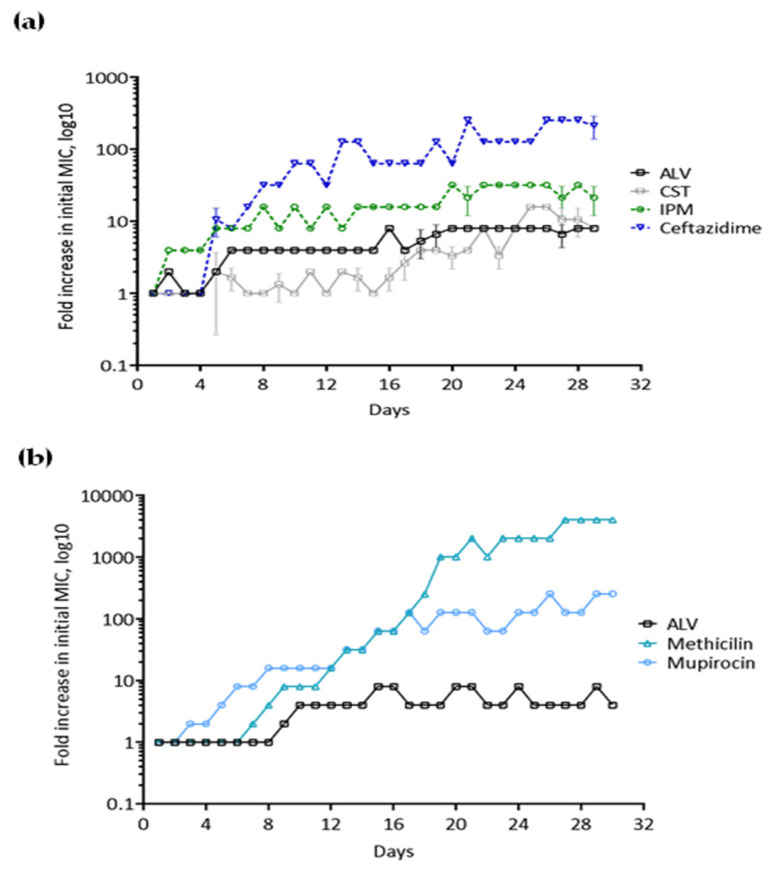
Resistance development in *P. aeruginosa* and *S. aureus* following prolonged exposure to ALV or reference antibiotics. (**a**,**b**) Monitoring of resistance induction in *P. aeruginosa* (**a**) and *S. aureus* (**b**) during one month of exposure to sublethal concentrations of ALV or reference antibiotics. MICs were determined daily, and resistance development is expressed as fold changes relative to the initial MIC.

Reference strains of *P. aeruginosa* (PAO1) and *S. aureus* (ATCC 29213) were exposed daily for 30 consecutive days to sublethal concentrations of ALV or antibiotics used in clinical practice (Figure 4).

A modest increase in MIC values (approximately 8-fold) was observed for both bacterial species following ALV exposure. In contrast, reference antibiotics (except CST) induced a much higher fold increase in MICs. In *P. aeruginosa*, MICs increased 21-fold with IPM and 213-fold with ceftazidime (Figure 4a). The resistance profile induced by ALV in *P. aeruginosa* was comparable to that observed with CST (Figure 4a), a cyclic AMP antibiotic that remains a last-resort therapeutic option against MDR Gram-negative strains. In *S. aureus*, resistance development was even more pronounced, with MIC values increasing up to 4069-fold under methicillin pressure (Figure 4b).

### 2.4. ALV Is Not Cytotoxic or Hemolytic at Bactericidal Concentrations

**Figure 5:**
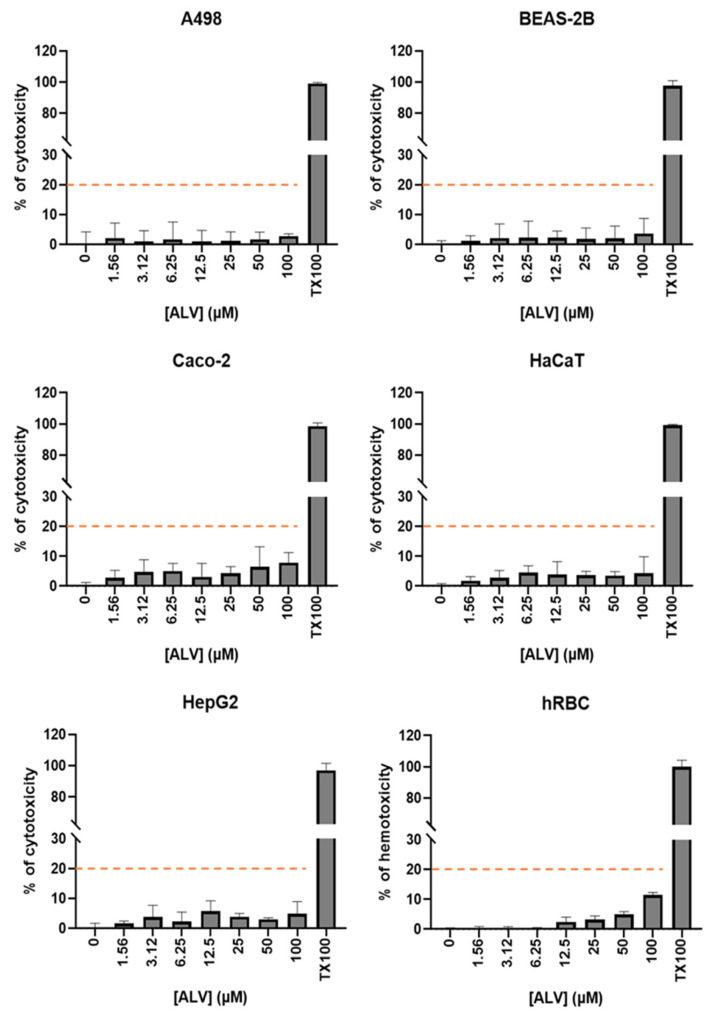
Cytotoxic and hemolytic effects of ALV on human cells. Toxicity of ALV on human cells was measured and expressed: (i) as percentage of cytotoxicity after treatment of A498, BEAS-2B, Caco-2, HaCaT, and HepG2 cells for 48 h with increasing concentrations of ALV, or (ii) as percentage of hemotoxicity after treatment of hRBC for 1 h with increasing concentration of peptide. Triton X100 at 0.1% was used as a positive control of cytotoxicity and hemotoxicity. The dashed line corresponds to 20% of cytotoxicity or hemotoxicity. Data represent the mean ± standard deviation (SD) from three independent experiments (*n* = 3).

Hemolysis assay using human red blood cells (hRBC) and cytotoxicity assay using human cells from the kidneys (A498), lungs (BEAS-2B), intestine (Caco-2), skin (HaCaT), and liver (HepG2) were performed across a range of ALV concentrations (Figure 5).

No significant cytotoxic effect of ALV was observed on human cells. Cytotoxicity of ALV remained below 20% at all concentrations tested, with the cell viability ranging from 92.13 to 97.18% at 100 µM of ALV. Consistently, hRBC hemolysis remained minimal (and below 20% hemolysis), reaching only 11.44% at 100 µM of ALV. These findings indicate that ALV exerts potent antibacterial activity at concentrations well below those associated with cytotoxicity or hemolysis.

### 2.5. ALV Preferentially Targets Bacterial Lipids

**Figure 6:**
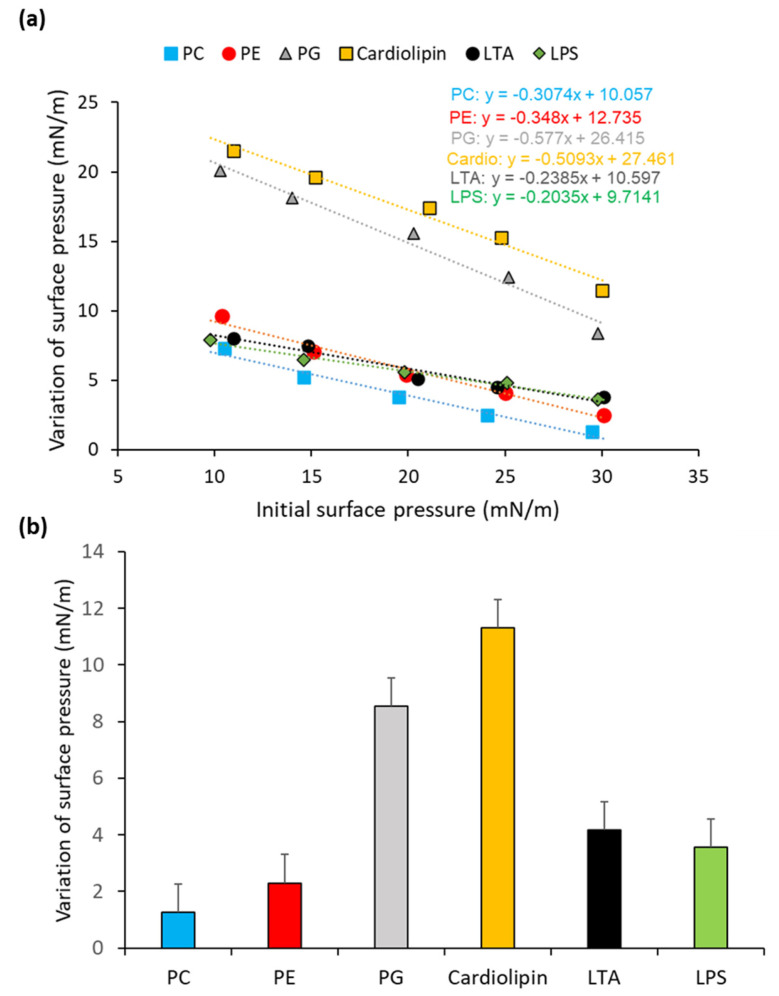
Insertion of ALV into lipid monolayers. Insertion of ALV into reconstituted monolayers composed of individual lipid components was analyzed using a Langmuir balance. Phosphatidylcholine (PC), phosphatidylethanolamine (PE), phosphatidylglycerol (PG), cardiolipin, lipoteichoic acid (LTA), and lipopolysaccharide (LPS) were tested. (**a**) Determination of the critical insertion pressure of ALV into each lipid monolayer. Changes in surface pressure induced by ALV insertion were measured at initial surface pressures ranging from 10 to 30 mN/m, and critical insertion pressures were calculated from the fitted equations. (**b**) Surface pressure variation induced by ALV at an initial surface pressure of 30 ± 0.5 mN/m, corresponding to the theoretical surface pressure of a biological membrane. Data are expressed as mean ± SD (*n* = 3).

The interaction of ALV with membrane lipids was assessed using a monolayer insertion assay (Figure 6).

The tested lipids represent the major components of human (eukaryotic) cell membranes, phosphatidylcholine (PC) and bacterial membranes, including phosphatidylethanolamine (PE), phosphatidylglycerol (PG), cardiolipin, lipoteichoic acid (LTA, for Gram-positive bacteria), and lipopolysaccharide (LPS, for Gram-negative bacteria).

Measurement of the critical insertion pressure (Figure 6a) revealed that ALV preferentially inserts into bacterial lipids rather than human lipids. The insertion threshold followed this descending order: cardiolipin (53.91 mN/m) > LPS (47.73 mN/m) > PG (45.77 mN/m) > LTA (44.43 mN/m) >> PE (36.59 mN/m) > PC (32.71 mN/m) (Table 3).

Consistently, the maximum change in surface pressure (Δπ) caused by ALV at an initial surface pressure of 30 ± 0.5 mN/m—mimicking the lateral pressure of biological membranes—was significantly greater for bacterial lipids compared to the human lipid PC (Figure 6b). The order of insertion efficacy was: cardiolipin (11.30 ± 0.43 mN/m) > PG (8.53 ± 0.41 mN/m) > LTA (4.16 ± 0.32 mN/m) > LPS (3.56 ± 0.55 mN/m) > PE (2.30 ± 0.20 mN/m) > PC (1.26 ± 0.25 mN/m). These biophysical data corroborate the results of antibacterial and cytotoxicity assays, confirming the selective affinity of ALV for bacterial membranes over human membranes, supporting its potential as a selective antibacterial agent.

### 2.6. Bactericidal Activity of ALV Is Redox-Independent in P. aeruginosa but Redox-Sensitive in S. aureus

**Figure 7:**
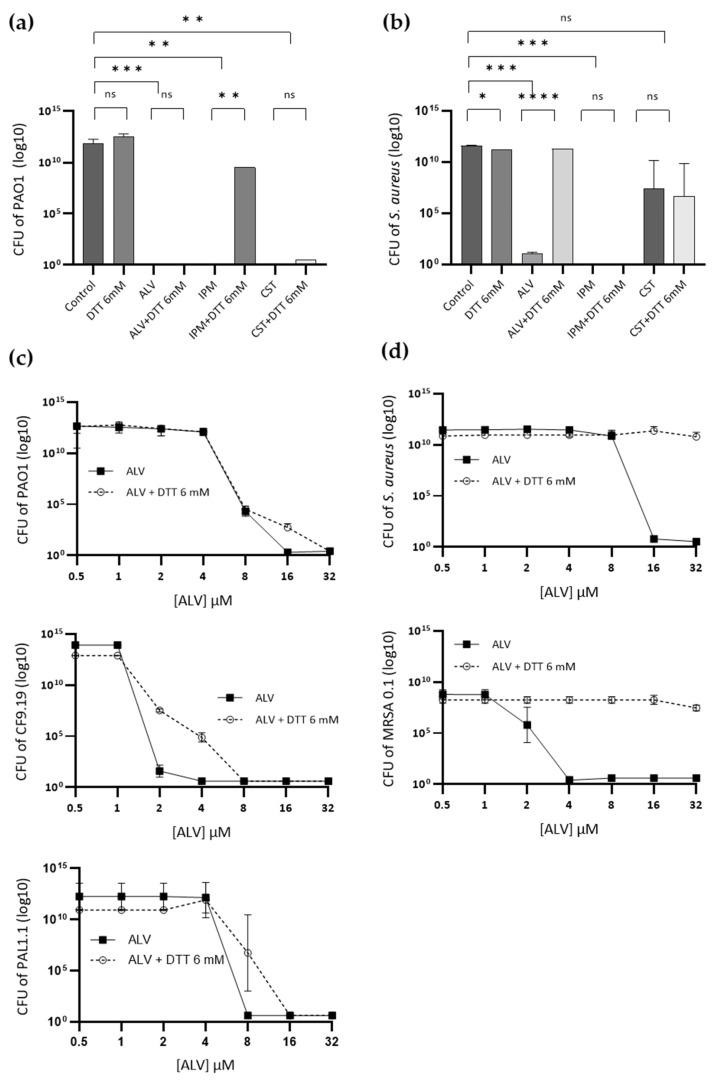
Effect of reducing conditions on the antibacterial activity of ALV. (**a**,**b**) *P. aeruginosa* (**a**) and *S. aureus* (**b**) were exposed to ALV at its MIC, as well as to the reference antibiotics CST and IPM, in the presence or absence of 6 mM DTT. Bacterial survival was determined by colony-forming unit (CFU) enumeration. (**c**,**d**) Dose–response killing curves of reference and clinical strains of *P. aeruginosa* (**c**) and *S. aureus* (**d**) following overnight exposure to ALV under reducing (+DTT, 6 mM) or non-reducing conditions. Each point represents an individual replicate (*n* = 3), and lines indicate the mean ± SD. Statistical analysis was performed using two-way ANOVA followed by Tukey’s multiple comparisons test to compare control and treated conditions, as well as the effect of DTT within each treatment. Significance levels are indicated as follows: *p* < 0.05 (*), *p* < 0.01 (**), and *p* < 0.001 (***); ns, not significant.

Colony-forming unit (CFU) counts of *P. aeruginosa* (Figure 7a) and *S. aureus* (Figure 7b) were determined after 24 h exposure to ALV or to reference antibiotics at therapeutic concentrations, including CST and IPM, under reducing conditions (6 mM DTT; see Section 4.3 Reducing Redox Environment). Control experiments confirmed that DTT alone did not influence bacterial growth.

In *P. aeruginosa*, the bactericidal activity of ALV was not affected by the redox modification, while IPM efficacy was markedly impaired and CST activity was slightly reduced (Figure 7a). By contrast, in *S. aureus*, ALV completely lost its antibacterial effect under reducing conditions (Figure 7b). Under the same conditions, IPM activity was only moderately reduced, and CST maintained its consistently low efficacy at the tested concentration.

Dose–response assays against reference and clinical strains of *P. aeruginosa* and *S. aureus* further corroborated these observations. In all the tested strains of *P. aeruginosa* (PAO1, CF9.19 and PAL1.1) (Figure 7c), ALV exhibited similar killing kinetics in the presence or absence of DTT. In contrast, in *S. aureus* (ATCC 29213 and MRSA) (Figure 7d), ALV activity was markedly altered under reducing conditions, showing a distinct profile compared to non-reducing conditions.

### 2.7. Antibiofilm Activities of ALV Under Reducing Conditions

**Figure 8:**
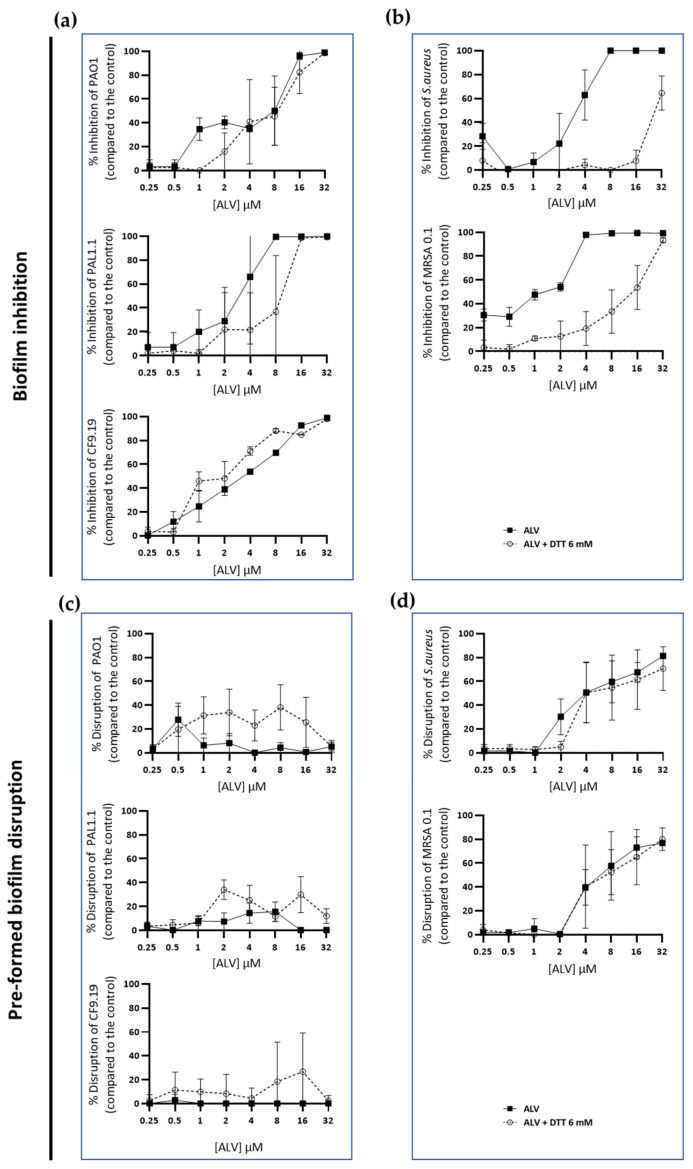
Antibiofilm activity of ALV under reducing conditions. (**a**,**b**) Inhibition of biofilm formation in reference and clinical strains of *P. aeruginosa* (**a**) and *S. aureus* (**b**) following ALV treatment under reducing (+DTT, 6 mM) and non-reducing conditions. (**c**,**d**) Disruption of preformed biofilms of reference and clinical strains of *P. aeruginosa* (**c**) and *S. aureus* (**d**) after ALV exposure under reducing (+DTT, 6 mM) and non-reducing conditions. In all panels, each point represents an individual replicate (n = 3), and lines indicate the mean ± SD. ALV maintained its disruptive effect on *S. aureus* biofilms (Figure 8d) in the presence of DTT, while no disruptive effect was still observed on *P. aeruginosa* biofilms (Figure 8c).

As shown in Figure 8, the inhibition of *P. aeruginosa* biofilm by ALV (Figure 8a) remains unaffected under reducing conditions, whereas the inhibition of *S. aureus* biofilm is altered by these conditions (Figure 8b). For both bacterial species, no significant differences in this response pattern were observed between the reference strains and the corresponding clinical isolates.

### 2.8. CD Analysis Reveals That DTT Addition Does Not Alter the Secondary Structure of ALV

**Figure 9:**
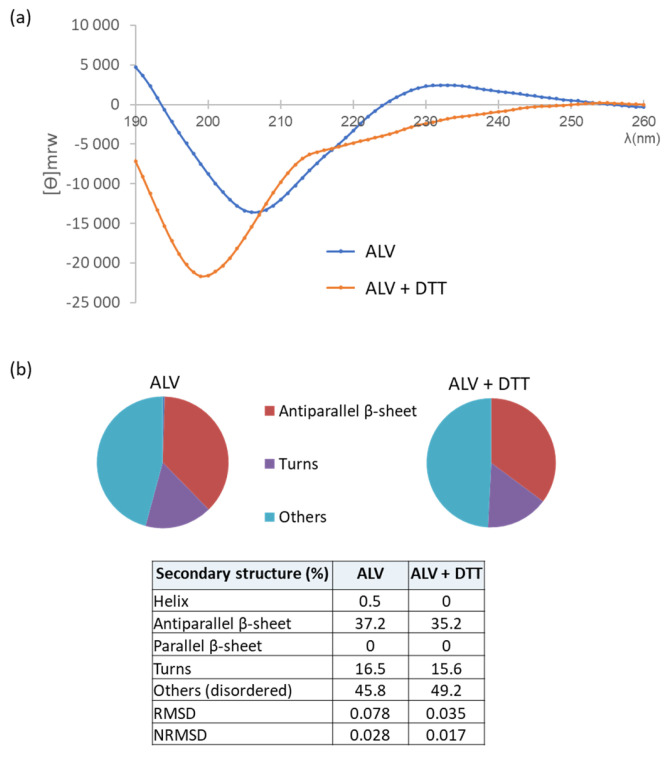
Far-UV CD spectra of ALV in the absence and presence of DTT. (**a**) CD spectra recorded between 190 and 260 nm. (**b**) BeStSeL deconvolution shows that ALV is mainly composed of antiparallel β-sheets w/o DTT treatment. The low Root Mean Square Deviation (RMSD) and normalized RMSD (NRMSD) values obtained for both fittings confirmed the reliability of the deconvolution.

CD spectra of ALV were recorded in the absence and presence of DTT to evaluate the impact of disulfide bond reduction on its secondary structure (Figure 9a,b). In both conditions, the spectra display a strong negative band around 200 nm, consistent with a structure dominated by β-sheets and disordered regions. In the absence of DTT, a moderate positive band is observed in the 225–230 nm region (Figure 9a), which disappears under reducing conditions. [22,23] Disulfide bonds can contribute to the CD spectra of polypeptides by producing a band at ~230 nm, the sign of which depends on the C–S–S–C dihedral angle. Typically, negative C–S–S–C angles, corresponding to a left-handed twist, give rise to a positive band at 230 nm. The positive band observed at 230 nm in the CD spectrum of ALV suggests that its disulfide bonds preferentially adopt a left-handed twist. This result is consistent with the reported NMR structure of ALV (PDB ID: 2LLR).

Spectral deconvolution (Figure 9b) using BeStSeL [24,25] revealed highly similar secondary structure compositions in both conditions. In the absence of DTT, ALV consists of approximately 37.2% antiparallel β-sheets, 16.5% turns, and 45.8% disordered structures, compared to 35.2%, 15.6%, and 49.2%, respectively, in the presence of DTT. The α-helical content remains negligible (<1%) in both cases. These findings indicate that ALV adopts a predominantly β-sheet structure. The similarity of the deconvolution profiles suggests that reduction of disulfide bonds does not significantly alter the global secondary structure of ALV.

### 2.9. ALV Confers Dose-Dependent Protection Against Acute P. aeruginosa Infection in the Galleria mellonella Model

**Figure 10:**
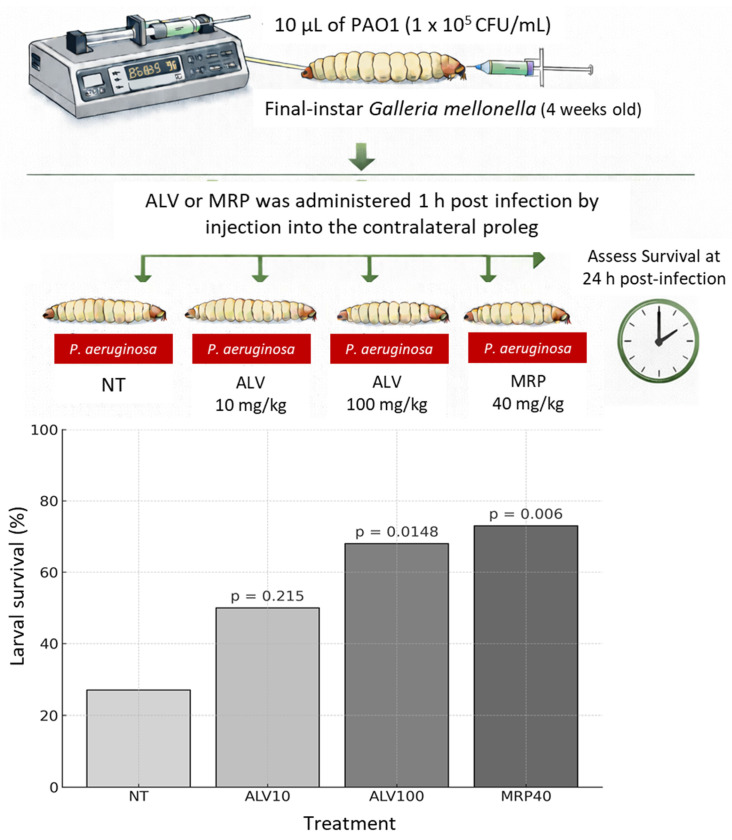
Protective efficacy of ALV against *P. aeruginosa* PAO1 infection in the *Galleria mellonella* model. Larvae were infected with *P. aeruginosa* PAO1 at an inoculum selected from the dose–response survival profile (1 × 10^5^ CFU/mL) and treated 1 h post-infection with ALV at 10 mg/kg (ALV10) or 100 mg/kg (ALV100). Meropenem (MRP) 40 mg/kg (MRP40) was used as a positive control for protection. Survival was assessed at 24 h post-infection and is expressed as the percentage of surviving larvae. Data represent a total of 22 larvae analyzed across three independent experiments. Statistical comparisons were performed using Fisher’s exact test by comparing each treated group with the untreated infected control (NT). Corresponding *p*-values are indicated above the bars.

We first assessed the virulence of *P. aeruginosa* strains PAO1, PAL1.1, and CF9.19 in the *Galleria mellonella* infection model (Figure S2 and Figure 10).

PAO1 caused a rapid decrease in larval survival within the first 24 h, indicating a high level of early virulence. PAL1.1 exhibited a delayed onset of lethality; however, its dose–response survival profile converged toward that of PAO1 at later time points. In contrast, CF9.19 did not induce detectable mortality across the range of inocula tested. Based on these virulence profiles, the highly virulent PAO1 strain was selected for subsequent experiments evaluating the protective effect of ALV against *P. aeruginosa* infection (Figure 10). Larval survival was monitored for up to 72 h. In this acute infection model, a significant effect of ALV was observed only at early time points, whereas no difference was detected at later stages compared with the infected untreated group. The 24 h time point was therefore retained as the most informative endpoint. The protective efficacy of ALV was next evaluated using the PAO1 strain at an infective dose selected from the dose–response survival model, corresponding to a condition yielding less than 25% larval survival at 24 h (Figure 10). Larvae were treated 1 h post-infection with ALV at 10 mg/kg or 100 mg/kg, and survival was assessed at 24 h post-infection, a time point capturing the acute phase of infection and the maximal discrimination between treatment conditions in this model. Compared with untreated infected larvae, treatment with ALV at 10 mg/kg resulted in a modest increase in survival that did not reach statistical significance. Treatment with ALV at 100 mg/kg led to a higher survival rate and a statistically significant improvement compared with untreated controls.

As a positive control, treatment with meropenem (40 mg/kg) significantly increased larval survival, confirming the responsiveness and validity of the infection model.

## 3. Discussion

Chronic infections caused by *P. aeruginosa* and *S. aureus* remain major clinical challenges in CF, largely due to antibiotic resistance, biofilm formation, and the altered physicochemical conditions of the CF lung [1,26]. The reducing and hypoxic microenvironment of CF airways not only promotes bacterial persistence but also compromises the efficacy of conventional antibiotics that rely on active bacterial metabolism or are sensitive to redox fluctuations [3,5]. In this context, the present study identifies the extremophile-derived peptide ALV, as a potent AMP capable of addressing these unmet therapeutic needs [14,27].

ALV exhibited strong, dose-dependent bactericidal activity against both *P. aeruginosa* and *S. aureus* pathogens, including multidrug-resistant clinical isolates. The identical MIC and MBC values across all tested strains confirm a primarily bactericidal mode of action. Permeabilization assays and scanning electron microscopy revealed extensive bacterial membrane damage consistent with a pore-forming mechanism, which likely explains both its rapid killing kinetics and the low propensity for resistance development. After 30 days of repeated exposure, ALV induced only a modest (eightfold) increase in MICs, comparable to CST—while conventional antibiotics such as IPM, ceftazidime, and methicillin triggered resistance increases exceeding 100- to 1000-fold.

The results of the lipid monolayer assays provide strong biophysical evidence that ALV displays a marked preference for bacterial membrane components over those found in human cells. Specifically, the higher insertion pressure thresholds and greater surface pressure variations in this positively charged AMP in the presence of bacterial lipids—such as cardiolipin, PG, LTA, and LPS—highlight its ability to interact more efficiently with the negatively charged and structurally distinct surfaces of bacterial membranes. In contrast, the much lower insertion into phosphatidylcholine (PC), a zwitterionic lipid predominantly found in eukaryotic membranes, suggests a reduced affinity for human cell membranes, which is consistent with the low cytotoxicity observed in parallel assays.

This lipid selectivity profile is consistent with the canonical mechanism of many AMPs [28], which exploit the marked differences in charge and lipid composition between host and microbial membranes to achieve selective targeting. ALV displays strong preferential insertion into cardiolipin and PG, two anionic lipids highly enriched in bacterial membranes, indicating that these components likely constitute primary docking and disruption sites. In addition, ALV interacts with LPS and LTA, which are surface-exposed in Gram-negative and Gram-positive bacteria, respectively, suggesting that membrane destabilization may be initiated at the outer leaflet prior to deeper membrane insertion. This membrane-active mode of action is further supported by parallels with arenicin, a β-hairpin AMP from *Arenicola marina*, which similarly inserts into bacterial membranes to form pores and induce rapid cell death [29]. Collectively, these observations support a pore-like, receptor-independent mechanism for ALV, driven by preferential interactions with anionic bacterial lipids and surface components, which underlies its potent antimicrobial activity while limiting host toxicity and the resistance development.

We next investigated whether ALV retained its antimicrobial efficacy under salt or redox conditions mimicking the *in vivo* environment during infection, particularly within the CF lung, which is characterized by a highly reducing environment (modeled here with 6 mM DTT) and elevated salt concentrations (300 mM NaCl). The activity of ALV against both bacteria was clearly salt-independent. Remarkably, ALV also preserved both its bactericidal activities under redox conditions against *P. aeruginosa*, whereas IPM efficacy was substantially reduced and CST was only moderately impaired. The differential effect of DTT on IPM and CST suggests a redox-dependent modulation of the Gram-negative envelope rather than a direct interaction with the antibiotics themselves, as neither molecule contains disulfide bonds and is therefore unlikely to be chemically modified by DTT.

To better understand the antibacterial activities observed under reducing conditions, we investigated whether DTT affected the structural organization of ALV (which contains 2 disulfide bonds) using CD spectroscopy. Analyses consistent with previous NMR data [14] confirmed a predominantly β-sheet/β-hairpin fold and showed that the secondary structure was largely preserved under reducing conditions. In the presence of DTT, ALV retained its anti-*P. aeruginosa* activity but lost activity against *S. aureus*, indicating that disulfide bonds are not strictly required for antimicrobial activity *per se*, but are important for target specificity. Similar structure–function decoupling has been described for several AMPs, where partial structural integrity remains sufficient for activity against some membranes but not others [30,31].

In Gram-negative bacteria such as *P. aeruginosa*, the outer membrane rich in LPS promotes strong electrostatic and amphipathic interactions with cationic peptides, which may not require a highly constrained tertiary structure. In this context, the preserved β-sheet scaffold of ALV which is a cationic AMP appears sufficient to maintain membrane-disruptive activity. By contrast, Gram-positive bacteria such as *S. aureus* possess a thick peptidoglycan layer and lack an outer membrane, which may impose stricter conformational requirements for peptide activity. Reduction of disulfide bonds may alter the spatial arrangement of key residues and impair efficient interaction with this type of cell envelope. Similar dependence on disulfide-stabilized topology has been reported for defensins which are also cysteine-rich AMPs, where reduction preferentially decreases activity against Gram-positive bacteria [31,32]. An alternative explanation is that DTT-reduced ALV may also be reoxidized by reactive oxygen species or other oxidizing systems produced by Gram-negative bacteria (and not by Gram positive), restoring its active oxidized form [33].

Overall, these results indicate that the β-sheet structure of ALV constitutes a robust structural framework sufficient for membrane interaction, while disulfide bonds fine-tune its three-dimensional conformation and optimize activity depending on bacterial envelope architecture. From an evolutionary perspective, the loss of anti-staphylococcal activity in reducing environments suggests that ALV may have evolved to function optimally within the physicochemical and microbial environment of its native habitat. Consistent with this, members of the genus *Pseudomonas* (and Gram-negative bacteria in general) are prevalent in the *A. pompejana* habitat, whereas bacteria of the genus *Staphylococcus* have not been described.

Because, in CF airways, biofilms contribute to long-term bacterial colonization, reduced antibiotic efficacy, and recurrent infections, we investigated the antibiofilm activity of ALV in the presence and absence of DTT. The inhibition of biofilm formation was consistent with the bactericidal activity observed for ALV under both reducing and non-reducing conditions. ALV efficiently inhibited biofilm formation in *P. aeruginosa* strains, including clinical isolates, and this activity was maintained under reducing conditions. In contrast, the inhibition of *S. aureus* biofilm formation by ALV was affected in a reducing environment. Interestingly, and specifically for *S. aureus* strains, ALV was able to disrupt preformed biofilms of both the reference strain and the clinical isolate in a redox-independent manner. Even though the mechanism behind these data remains to be elucidated [10], the disruptive effect of ALV on established biofilms highlights an additional therapeutic advantage, particularly in the context of chronic and persistent infections where mature biofilms are difficult to eradicate.

In this context, we investigated whether the robust *in vitro* properties of ALV could translate into measurable *in vivo* protection using the *Galleria mellonella* acute infection model. This invertebrate model represents an ethical and experimental preliminary step before progressing to mammalian studies, allowing early evaluation of antimicrobial efficacy while limiting the use of vertebrate animals. Under the experimental conditions tested, ALV treatment led to a dose-dependent improvement in larval survival following *P. aeruginosa* infection. Although this protective effect was more modest than that achieved with the antibiotic control meropenem, which consistently conferred strong protection and thereby validated the infection model, the observed survival benefit supports a biologically relevant *in vivo* activity of ALV. Importantly, the limited magnitude of protection in this acute infection setting may reflect the context-dependent nature of ALV’s antimicrobial efficacy and suggests that its full potential could be underestimated in models that do not recapitulate the chronic, biofilm-associated conditions characteristic of CF infections.

Natural antimicrobial molecules, particularly AMPs, represent a rich and largely untapped reservoir for therapeutic innovation [34]. Few studies have demonstrated that AMPs or nature-inspired analogs can retain activity in hostile environments resembling CF sputum, where pH, ionic strength, and protease activity undermine the stability and efficacy of traditional antibiotics [35]. Frog-skin–derived peptides, for instance, significantly reduced bacterial burden in murine lung infection models of *P. aeruginosa*, demonstrating the translational feasibility of peptide-based therapies for respiratory infections. Overall, the accumulating body of evidence supports the potential of natural and bioinspired peptides [27] as valuable complements to conventional antibiotics in the treatment of infections [6].

Among these candidates, the present data identify ALV as particularly promising because of its excellent safety profile, which represents a major advantage over other AMPs such as CST, whose clinical use is limited by nephrotoxicity. No cytotoxicity was detected, even at ALV concentrations well above bactericidal levels, including on BEAS-2B human bronchial epithelial cells, which is particularly relevant for CF airways. These data strongly suggest that ALV is suitable for inhalation *via* nebulization as a potential treatment for CF pulmonary infections, where repeated direct exposure of the airway epithelium to the peptide is expected. In addition, ALV retains its antibacterial activity under reducing conditions, in the presence of salt, and after incubation in human serum, indicating good stability and preservation of function under physiologically relevant conditions. Its activity across a wide pH range, previously demonstrated, further supports its robustness in variable host environments. ALV also exhibits antibiofilm activity, a property that is uncommon among AMPs, and does not induce bacterial resistance. The promising results obtained in the *Galleria mellonella* infection model further highlight its therapeutic potential and support its continued preclinical development. Altogether, these findings reinforce the rationale for advancing toward *in vivo* evaluation in murine infection models. Future work will focus on better characterizing key pharmacological parameters, including the determination of the maximum tolerated dose and toxic dose, comprehensive toxicity profiling, ADME properties (absorption, distribution, metabolism, and excretion), pharmacokinetics, and biodistribution. Additional studies under more complex CF-associated conditions, particularly in the presence of proteases, neutrophil elastase, mucins, or even CF sputum, also represent important perspectives to further strengthen the therapeutic potential of ALV.

## 4. Materials and Methods

### 4.1. ALV Synthesis

ALV (Alvinellacin-NH2; sequence: RGC1YTRC2WKVGRNGRVC3MRVC4T-NH2; disulfide bonds C1–C4 and C2–C3) was synthesized by standard solid-phase peptide synthesis (SPPS) (GENEPEP, Hérault, France). Analytical characterization was performed by UPLC–MS using an Ultimate 3000 UPLC system (Thermo Fisher, Waltham, Franklin, USA) equipped with a BEH C18 column (Milford, MA, USA) (300 Å, 1.7 µm, 2.1 × 150 mm), diode array detection, and an LCQ Fleet ion-trap mass spectrometer. Samples were analyzed at 50 °C using a linear 0–70% CH3CN/0.1% TFA gradient over 20 min at 0.4 mL·min^−1^. UV detection was carried out at 215, 254 and 280 nm. MS data were acquired in ESI+ mode (m/z 300–2000) (Figure S1a). MALDI-TOF mass spectra of purified peptides were obtained on a Bruker Autoflex Speed instrument (Billerica, MA, USA) using CHCA or SA matrices (10 mg·mL^−1^ in 0.1% TFA, H2O/CH3CN 1:1 *v*/*v*). Reported m/z values correspond to monoisotopic ions unless specified otherwise (Figure S1b).

### 4.2. CD Analysis of ALV w/o DTT

The concentration of synthetic ALV solubilized in 10 mM sodium phosphate buffer (pH 7.2) was determined by UV absorbance at 280 nm using a NanoDrop spectrophotometer (Wilmington, DE, USA), based on its theoretical extinction coefficient (ε = 7240 L·mol^−1^·cm^−1^). For CD measurements, the stock solution was diluted to 0.07 mg·mL^−1^ in 10 mM sodium phosphate buffer (pH 7.2) containing DTT (1 mM final concentration) or not. All the solutions used for CD measurements were prepared in a nitrogen glove box (O2 < 0.1 ppm) to minimize thiol oxydation. The use of 6 mM DTT precluded CD analysis due to strong absorbance below 200 nm. CD spectra were recorded on a Jasco J-815 spectropolarimeter ( Hachioji, Tokyo 193-0835, Japan) equipped with a PFD-425S Peltier temperature control unit, using a 0.1 cm path-length quartz cuvette (Hellma, Müllheim, Germany). Spectra were acquired between 185 and 260 nm at 25 °C with a bandwidth of 2 nm, a data pitch of 1 nm, and an integration time of 1 s. Each spectrum corresponded to the average of ten accumulations and was smoothed using a five-point algorithm. CD data were expressed as mean residue weight ellipticity.

CD spectra were deconvolved using BeStSeL webserver.1 [24].

### 4.3. Reducing Redox Environment

A reducing redox environment was established with DTT at a final concentration of 6 mM. At this concentration, and under neutral pH (pH 7.0), the redox potential (Eh) is estimated to be approximately −330 mV. DTT was stored in the solid form in a glove box under nitrogen to prevent oxidation, and the solution was prepared freshly before each experiment to ensure maximal reducing capacity.

### 4.4. Bacterial Strains

Reference bacterial strains used in this study were obtained from the American Type Culture Collection (ATCC) and included *P. aeruginosa* (PAO1, ATCC 27853) and *S. aureus* (ATCC 29213). Clinical strains used were (i) the methicillin-resistant *S. aureus* strain, MRSA 0.1, isolated from a blood culture of a patient hospitalized in geriatrics care, (ii) the *P. aeruginosa* CF 9.19 isolated from the sputum of CF patient (National Reference Centre for Antibiotic Resistance, university hospital of Besançon, France) and (iii) the *P. aeruginosa* PAL1.1 that was isolated from the airways of an intensive care unit (ICU) patient (Lille CHU hospital, France), with ventilator-associated pneumonia. A detailed characterization of the antibiotic susceptibility profiles of PAO1, PAL1.1 and CF9.19 is provided in Table S1.

### 4.5. Antibacterial Activity Assays

The antibacterial activity was tested against both reference and clinical strains. For *P. aeruginosa*, reference antibiotics used were: (i) CST sulfate (European Pharmacopoeia), a last-resort polymyxin, widely used clinically to treat severe multidrug-resistant *P. aeruginosa* infections, including healthcare-associated pneumonia (ii) IPM, a broad-spectrum carbapenem, is prescribed as a first-line therapy for serious *P. aeruginosa* infections and iii) ceftazidime, a third-generation cephalosporin, used to manage *P. aeruginosa* infections, particularly during acute pulmonary exacerbations in CF patients or in immunocompromised individuals. For *S. aureus*, methicillin was used as a phenotypic marker distinguishing MSSA from MRSA strains, guiding the use of appropriate agents such as vancomycin or linezolid in resistant cases. Mupirocin was also tested, as it is used as a topical antibiotic and routinely applied for nasal decolonization of *S. aureus* carriers, especially preoperatively or in patients at heightened risk of nosocomial infection.

MIC and MBC were determined using the broth microdilution method in 96-well plates as previously described [15]. All assays were performed in triplicate. MIC and MBC were also measured in the presence of NaCl (at 150 and 300 mM final concentration) or after incubation of ALV with 50% (v:v) of human serum (from Sigma Aldrich, city, state, country) at 37 °C during 4 h

### 4.6. Time-Kill Assay

Kinetic activity of bactericidal activity was assessed against *P. aeruginosa* and *S. aureus* [36]. Cultures in exponential phase (10^6^ CFU/mL) in Mueller–Hinton broth (MHB; Difco) were treated with serial concentrations of ALV (0.25–16 μM) in 96-well plates (CytoOne^®^, STARLAB). OD_600_ was recorded every hour over 20 h at 37 °C with shaking (140 rpm) using a Tecan Sunrise microplate reader.

Dose-dependent killing was also measured. Bacteria (10^6^ CFU/mL) were incubated overnight with increasing ALV concentrations (4, 8, 12, 16 μM). Viability was assessed by CFU counting after plating on MHB agar and 48 h incubation at 37 °C [37]. The impact of DTT was assessed by comparing +DTT and −DTT conditions using the same approach. Statistical analyses were conducted with GraphPad Prism 10, using a significance threshold of α = 0.05. Comparisons between control (no treatment) and treated conditions (peptide or antibiotics) were performed using the Mann–Whitney U test.

### 4.7. Scanning Electron Microscopy (SEM)

Bacteria were grown to the exponential phase, diluted by 1:100 in MHB, and incubated for 2 h at 37 °C. A total of 50 μL of bacterial suspension was incubated with 5 μL of peptides (50 μM) or deionized water (control) for 4 h at 20 °C. Samples were fixed with 2.5% glutaraldehyde, centrifuged onto poly-lysine-coated slides (SHANDON 3, 1000 rpm, 8 min), sputter-coated with 5 nm platinum (Quorum Q150T ES), and imaged with a Merlin VP Compact Zeiss SEM.

### 4.8. Bacterial Membrane Permeabilization Assay

The effect of ALV on bacterial membrane integrity was evaluated through measurement of the release of intracellular ATP in culture media, as previously described [38]. Log-phase bacterial suspensions of *P. aeruginosa* (PA01) or *S. aureus* (ATCC 29213) were prepared in MH media from overnight liquid cultures as explained above. Bacteria were diluted at a density of 10 × 10^7^ bacteria per mL of MH containing ATP detection reagent based on luciferase (Yelen, France) into 96-well white plates for luminescence (Greiner BioOne) and exposed to concentrations of ALV corresponding to ¼ of MIC to 8 times MIC (1/2 dilutions). After 1, 5, 10, 20, 40, and 80 min of incubation at 37 °C, the luminescence of the wells was measured using a microplate reader (Biotek, Synergy Mx). Untreated conditions correspond to bacteria treated with vehicle alone. Lysis buffer provided in the ATP detection kit was used as a positive control, giving 100% bacterial membrane permeabilization. Experiments were conducted in triplicate (n = 3).

### 4.9. Antibiofilm Assays

Antibiofilm activity [39] was evaluated against the reference and clinical isolates of *P. aeruginosa* and *S. aureus*. Biofilm-forming ability was classified according to the crystal violet assay using the Optical Density (OD) of the untreated control biofilm for each strain. The cut-off value (ODc) was calculated as: ODc = meanOD negative control +3× Standard Deviation. Strains were then classified as weak, moderate or strong biofilm producers according to [40]. Based on this classification, PAO1, *S. aureus* ATCC 29213 and MRSA 0,1 were moderate biofilm formers, whereas PAL1.1 and CF9.19 were strong biofilm formers.

Overnight cultures in LB broth were diluted to an OD_580_ of 0.08. Then, 200 µL of suspension was added to sterile 96-well polystyrene microplates and incubated statically at 37 °C for 24 h to allow biofilm formation. ALV was applied either concurrently with inoculation (preventative assay: growth inhibition of the biofilm) or after the 24-h biofilm formation period (therapeutic assay: disruption of the pre-formed biofilm), at various concentrations starting at 0.25, 0.5, 1, 2, 4, 8, 16 and 32 µM. The subinhibitory concentrations were ≤4 µM for *P. aeruginosa* and ≤8 µM for *S. aureus*.

Plates were further incubated for 24 h. Following treatment, wells were gently rinsed three times with phosphate-buffered saline (PBS) to remove planktonic cells. Biofilms were fixed in methanol for 15 min, stained with 0.1% crystal violet for 15 min, and then rinsed thoroughly. The biofilm-associated dye was dissolved in 95% ethanol, and absorbance was measured at 570 nm. Planktonic growth controls were performed in parallel under the same conditions, with bacterial growth monitored at OD_600_.

### 4.10. Induction of Resistance

*P. aeruginosa* PAO1 or *S. aureus* ATCC 29213 cultures were exposed daily for 30 days to ALV or reference antibiotics. MICs were determined by standard broth microdilution on day 1. Each day, the culture from the highest antibiotic concentration allowing growth was diluted 1:100 in MHB to initiate the next passage. Wells without growth were plated on MHA with high ALV concentrations (80 and 160 µM) to detect resistant clones. Experiments were conducted at pH 7.

### 4.11. Toxicity Assay Using Human Cells

Cytotoxicity of ALV was assessed as previously described [41] using human kidney (A498; ATCC^®^ HTB-44™), lung (BEAS-2B; ECACC), intestinal (Caco-2; ATCC^®^ HTB-37™), skin (HaCaT; Creative Bioarray), and liver cells (HepG2; ATCC^®^ HB-8065™). Cells were maintained in DMEM supplemented with 10% FBS, 1% L-glutamine, and 1% penicillin–streptomycin, and incubated at 37 °C with 5% CO_2_. After detachment with trypsin–EDTA and counting, cells were seeded in 96-well plates at ~10,000 cells/well. Once confluent (48 h), media were replaced, and cells were exposed to increasing ALV concentrations. After 48 h incubation, viability was quantified by resazurin (30 µg/mL in PBS++), with fluorescence measured at 530/590 nm. Data were normalized to untreated controls; 0.1% Triton X-100 served as a cytotoxicity control (100% toxicity). Experiments were performed in triplicate (n = 3).

### 4.12. Hemotoxicity Evaluation

Hemolytic activity of ALV was evaluated using human red blood cells (hRBCs; Divbio Science Europe) as previously reported (DOI: 10.1038/s41522-022-00320-0). After three PBS washes and centrifugation (800× *g*, 5 min), hRBCs were resuspended at 8% in DMEM without phenol red supplemented with 10% FBS. A total of 150 µL of hRBCs was added to 96-well plates containing 150 µL of ALV at increasing concentrations. Following 1 h incubation at 37 °C, samples were centrifuged (800× *g*, 5 min) and 100 µL of supernatant was transferred to fresh plates. Hemolysis was quantified at 450 nm. Triton X-100 (0.1% *v*/*v*) was used as a positive control (100% hemolysis). All experiments were performed in triplicate (n = 3).

### 4.13. Peptide–Lipid Interaction Assay

The interaction of ALV with lipids was assessed using a lipid monolayer assay as previously described [42]. Prokaryotic and eukaryotic lipids (cardiolipin, POPC, POPE, POPG; Avanti Polar Lipids) as well as LTA (*S. aureus*) and LPS (*P. aeruginosa*) (Invitrogen) were tested. Lipids were prepared at 1 mg/mL in chloroform:methanol (2:1) and stored under nitrogen at −20 °C. Monolayers were formed at the air–water interface (PBS) by spreading lipids to the desired initial surface pressure. After 5–10 min stabilization, 8 µL of ALV were injected into the subphase to reach a final concentration of 1 µM, previously identified as optimal for insertion. Surface pressure changes induced by peptide insertion were continuously monitored until equilibrium using an automated microtensiometer (µTROUGH SX, Kibron). The critical insertion pressure (CIP) was determined by measuring peptide-induced pressure increases at initial surface pressures between 10 and 30 mN/m and extrapolating the initial pressure corresponding to zero insertion. ALV insertion was also evaluated at 30 ± 0.5 mN/m, a value representative of the outer leaflet of biological membranes. Experiments were performed at 20 ± 1 °C and analyzed using Firmware 2.5 (Kibron). The accuracy of surface pressure measurements under our conditions was ±0.25 mN/m.

### 4.14. In Vivo Evaluation of the ALV Activity Using the Galleria mellonella Larval Model

Final instar *Galleria mellonella* larvae (four weeks old; 220–300 mg) were used for all *in vivo* experiments [43,44]. Larvae originated from a genetically homogeneous colony maintained for more than 20 years and were supplied by the Microbial Genetics and Environment team at the Micalis Institute (INRAE). Injections were performed using a microinfusion system consisting of a 27-gauge microperfuser (Venofix^®^ 27G, B. Braun) mounted on a 1 mL syringe (BD Plastipak™ Tuberculin, ref. 00655) and driven by a syringe pump (KD Scientific) to ensure accurate and reproducible injection volumes. Bacterial suspensions or control solutions (10 µL) were injected into the hemocoel *via* the last left proleg. For each experiment, a group of non-infected larvae injected with phosphate-buffered saline (PBS) was included as an injection control to monitor procedure-related mortality and to verify the absence of toxicity associated with the injected volumes. Ten larvae were used per condition unless otherwise specified

To assess the virulence of *P. aeruginosa* strains PAO1, PAL1.1, and CF9.19, groups of ten larvae were infected with increasing bacterial inocula ranging from 10^1^ to 10^7^ CFU/mL. Larvae were incubated at 37 °C, and survival was monitored daily for three days post-infection. Dose–response survival data were analyzed using logistic regression to model the relationship between bacterial dose and larval survival. A total of 15 independent experiments, each comprising ten larvae per condition, were included in the dose–response analysis.

Prior to infection experiments, the toxicity of ALV was assessed in *Galleria mellonella*. Larvae (n = 10 per condition) were injected with increasing concentrations of ALV (5, 10, 20, 50 and 100 mg/kg) and monitored over 72 h. No mortality or signs of melanization were observed at any tested dose. The highest concentration tested corresponded to the maximal injectable dose (10 µL of a 10 mM stock solution).

ALV efficacy was evaluated using the *P. aeruginosa* reference strain PAO1. Based on the analysis of the dose–response survival profile, an infective dose of 1 × 10^5^ CFU/mL was selected, corresponding to a condition yielding less than 25% larval survival at 24 h. This experimental setting ensured a robust infection while allowing the detection of potential treatment effects. ALV was administered 1 h post-infection at doses of 10 mg/kg or 100 mg/kg by injection into the contralateral proleg to avoid interference with the infection site. Meropenem (40 mg/kg) was used as a positive control for protection. Untreated infected larvae and PBS-injected larvae served as negative and injection controls, respectively. Larval survival was assessed at 24 h post-infection. Experiments were performed in three independent replicates, with 10 larvae in one experiment and 6 larvae in each of the two others.

## 5. Conclusions

ALV illustrates the potential of extremophile-derived peptides as promising templates for next-generation therapeutics designed to function in hypoxic, biofilm-rich, and redox-imbalanced environments. Ongoing efforts are now focused on validating these effects through *in vivo* approaches in murine models, which will be essential to confirm their therapeutic potential and support future development in CF and other chronic respiratory infections.

## Figures and Tables

**Table 1:** In vitro antibacterial activity of ALV expressed as minimum inhibitory concentration (MIC) and minimum bactericidal concentration (MBC) values (µM) against ATCC reference (Ref) and clinical strains of *P. aeruginosa* and *S. aureus* in µM and in mg/L (in brackets). Abbreviation: NA, not active.

	*P. aeruginosa*						*S. aureus*			
	Ref PAO1		PAL1.1		CF9.19		Ref ATCC29213		MRSA	
	MIC	MBC	MIC	MBC	MIC	MBC	MIC	MBC	MIC	MBC
ALV	4 (10.4)	4 (10.4)	4 (10.4)	8 (20.8)	2 (5.2)	2 (5.2)	8 (20.8)	8 (20.8)	2 (5.2)	4 (10.4)
ALV + DTT 6 mM	8 (20.8)	8 (20.8)	8 (20.8)	16 (41.6)	4 (10.4)	4 (10.4)	>32 (>83.2)	>32 (>83.2)	>32 (>83.2)	>32 (>83.2)
CST	0.13 (0.25)	0.13 (0.25)	<0.45 (<0.5)	<0.45 (<0.5)	<0.45 (<0.5)	<0.45 (<0.5)	1.7 (2)	6.9 (8)	>13.8 (>16)	>13.8 (>16)
CST + DTT 6 mM	0.52 (1)	0.52 (1)	<0.45 (<0.5)	<0.45 (<0.5)	<0.45 (<0.5)	<0.45 (<0.5)	1.7 (2)	6.9 (8)	>13.8 (>16)	>13.8 (>16)
IPM	6.7 (2)	6.7 (2)	26.7 (8)	53.4 (16)	13.4 (4)	26.7 (8)	<0.03 (<0.25)	0.8 (0.12)	0.8 (0.12)	0.8 (0.12)
IPM + DTT 6 mM	106.8 (32)	213.8 (64)	53.4 (16)	106.8 (32)	53.4 (16)	53.4 (16)	0.8 (0.12)	>26.7 (>8)	26.7 (8)	26.7 (8)
DTT 6 mM	NA	NA	NA	NA	NA	NA	NA	NA	NA	NA

**Table 2:** Impact of NaCl and human serum on antibacterial activity of ALV expressed as MIC and MBC values (µM) and in mg/L (in brackets) against *P. aeruginosa* (PA01) and *S. aureus* (ATCC 29213). MH: culture medium.

	MH		MH + NaCl 150 mM		MH + NaCl 300 mM		MH + Human Serum 50%	
	MIC	MBC	MIC	MBC	MIC	MBC	MIC	MBC
*P. aeruginosa* (PA01)	4 (10.4)	4 (10.4)	4 (10.4)	4 (10.4)	4 (10.4)	8 (20.79)	4 (10.4)	4 (10.4)
*S. aureus* (ATCC 29213)	8 (20.79)	8 (20.79)	8 (20.79)	8 (20.79)	8 (20.79)	8 (20.79)	8 (20.79)	8 (20.79)

**Table 3:** Lipid insertion values. Critical pressures of insertions and variations in surface pressure caused by the insertion of ALV into lipid monolayers were calculated from Figure 5.

Lipids	PC	PE	PG	Cardiolipin	LTA	LPS
Critical pressure of insertion (in mN/m)	32.71	36.59	45.77	53.91	44.43	47.73
Variation in surface pressure at an initial pressure of 30 mN/m (in mN/m)	1.26 ± 0.25	2.30 ± 0.20	8.53 ± 0.41	11.30 ± 0.43	4.16 ± 0.32	3.56 ± 0.55

## Data Availability

All data generated or analysed during this study are included in this published article and its Supplementary Information Files. Additional materials are available from the corresponding author upon reasonable request.

## Supplementary Materials

The following supporting information can be downloaded at https://www.mdpi.com/article/10.3390/md24050164/s1: Table S1: Antibiotic susceptibility of *P. aeruginosa* PAO1, PAL1.1 and CF9.19; Table S2: Antibiotic susceptibility of *S. aureus* ATCC 29213 and MRSA 0.1; Figure S1: Purification and mass spectrometry analysis of ALV; Figure S2. Dose–response survival analysis and LD_50_ determination in *Galleria mellonella*.
