# Supplementary material for: Bioinspired Antimicrobial Strategy: An Extremophile Deep Sea Peptide to Combat Cystic Fibrosis Infections Caused by Pseudomonas aeruginosa and Staphylococcus aureus
Source: Mar Drugs. 2026 May 5;24(5):164. doi: 10.3390/md24050164 (PMC13208616; doi:10.3390/md24050164)

**SUPPLEMENTARY DATA FOR**

# Bioinspired Antimicrobial Strategy: An Extremophile deep sea peptide to combat Cystic Fibrosis infections caused by *Pseudomonas aeruginosa* and *Staphylococcus aureus*

**Table S1: Antibiotic susceptibility of *P. aeruginosa* PAO1, PAL1.1 and CF9.19**

MICs were determined by broth microdilution for a panel of antibiotics used against *P. aeruginosa*. For the three strains, colistin (CST) MIC values were ≤2 mg/L, corresponding to susceptible with increased exposure (S–I) according to current CLSI recommendations. PAO1 was susceptible (S) to all other tested antibiotics. PAL1.1 was resistant (R) to ceftazidime, meropenem, gentamicin, and ciprofloxacin, and showed intermediate susceptibility to imipenem. CF19 was resistant to ceftazidime, gentamicin, and ciprofloxacin, and showed intermediate susceptibility to imipenem and meropenem. Values correspond to three biological replicates performed in two independent repetitions.

| Antibiotics | MIC (mg/L) / strains | | |
|---|---|---|---|
| | PAO1 | PAL1.1 | CF19 |
| Colistin (CST) | 2 (S-I) | 1 (S-I) | 0.5 (S-I) |
| Ceftazidime (CAZ) | 2 (S) | 64 (R) | >128 (R) |
| Imipenem (IPM) | 2 (S) | 8 (I) | 4 (I) |
| Meropenem (MEM) | 2 (S) | 32 (R) | 4 (I) |
| Gentamicin (GEN) | 2 (S) | >128 (R) | 64 (R) |
| Ciprofloxacin (CIP) | 0.25 (S) | 32 (R) | 4 (R) |

**Table S2: Antibiotic susceptibility of *S. aureus* ATCC 29213 and MRSA 0.1**

MICs were determined by broth microdilution for a panel of antibiotics used against *S. aureus*. Values correspond to three biological replicates performed in two independent repetitions.

| Antibiotics | MIC (mg/L) / strains | |
|---|---|---|
| | ATCC 29213 | MRSA 0.1 |
| Penicillin (PCN) | <0,25 | >0,25 |
| Kanamycin (KAN) | <0,25 | >32 |
| Tobramycin (TOB) | <0,25 | >8 |
| Gentamicin (GEN) | <0,5 | <0,5 |
| Tetracycline (TET) | <1 | <1 |

| | | |
|---|---|---|
| Trimethoprim–Sulfamethoxazole (Bactrim) SXT | <10 | <10 |
| Ofloxacin (OFX) | <0,5 | <0,5 |
| Erythromycin (ERY) | <0,5 | 1 |
| Vancomycin (VAN) | 1 | 1 |

**Figure S1: Purification and mass spectrometry analysis of ALV. (a)** UPLC chromatogram of ALV purification using a C18 BEH 300 Å column. **(b)** ESI-MS spectrum of the purified peptide acquired in positive ion mode. **(c)** MALDI-TOF-MS spectrum of ALV acquired in positive reflectron mode using CHCA as the matrix.

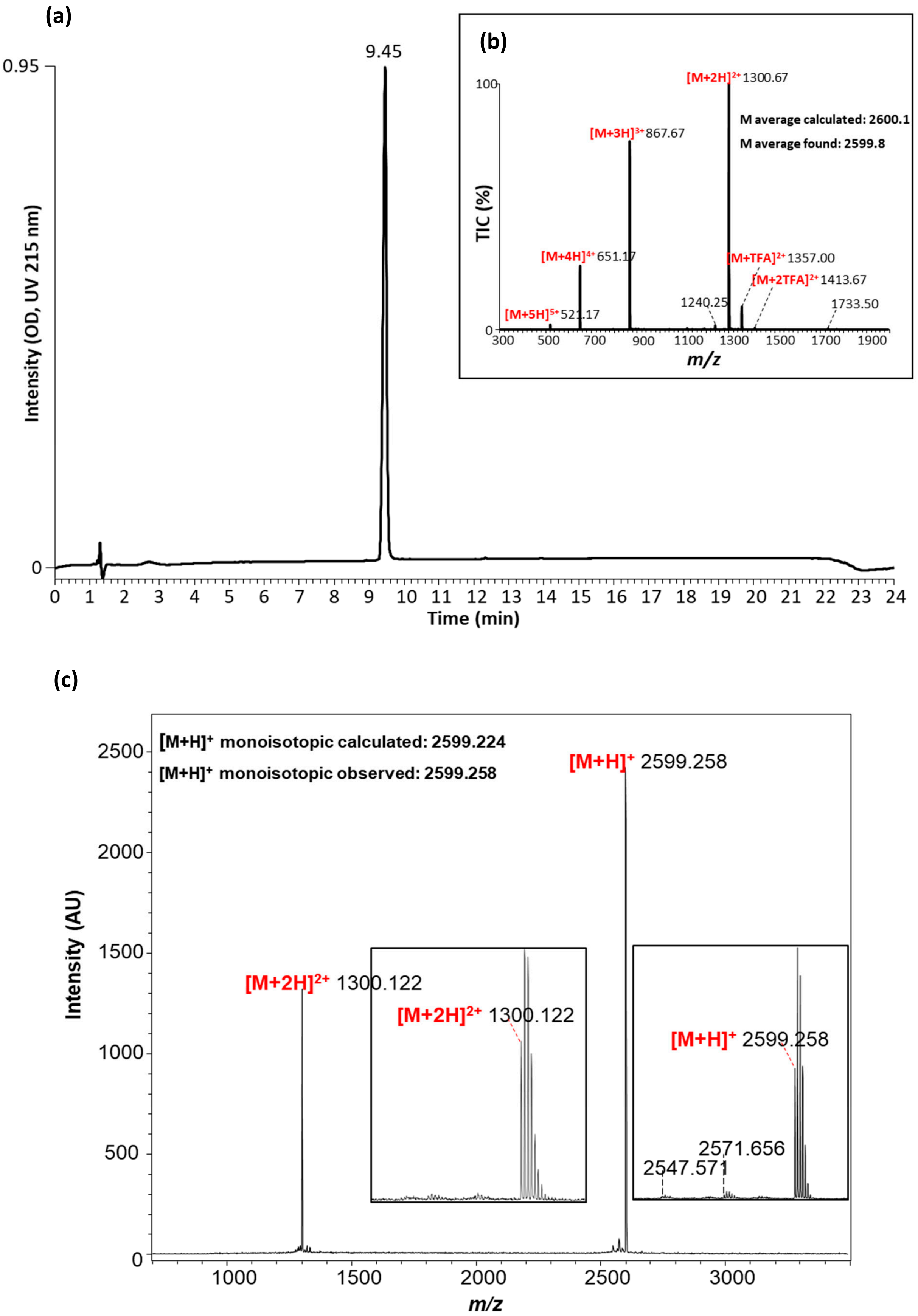

**Figure S2. Dose–response survival analysis and $LD_{50}$ determination in *Galleria mellonella*.** Dose–response survival curves for larvae infected with increasing inocula of strains PAO1 and PAL1.1, monitored over a 3-day period. Survival data at days 1, 2, and 3 were fitted using a generalized linear model (GLM) with a logistic link applied to $log_{10}$-transformed bacterial loads. The horizontal line indicates 50% survival, and dashed vertical lines denote $LD_{50}$ estimates with corresponding 95% confidence intervals. CF9.19 was not included in this analysis, as it did not induce detectable mortality in this model.

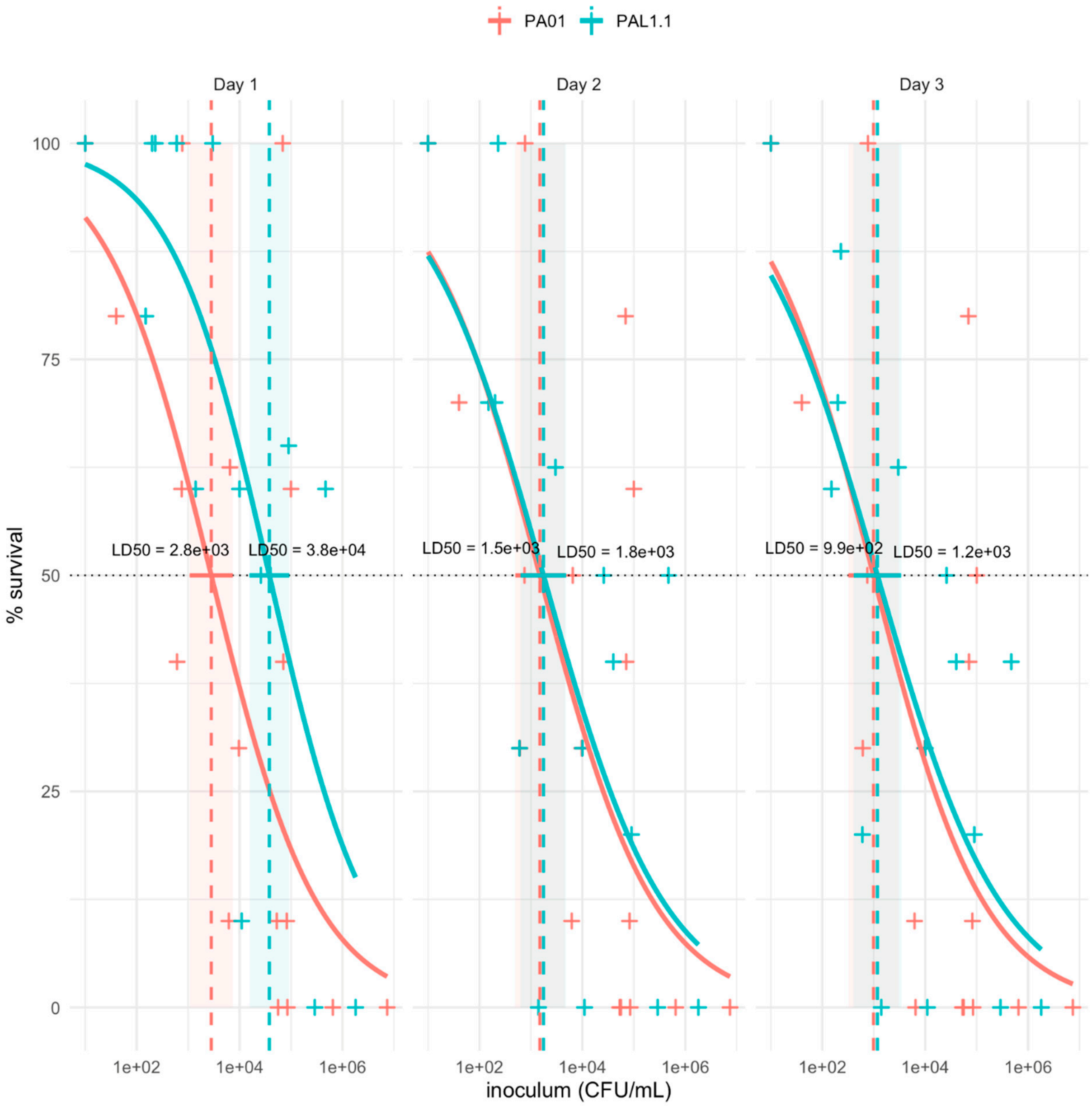

Supplement: Supplementary file 1 [file marinedrugs-24-00164-s001.zip › marinedrugs-4235170-supplementary.pdf]
